# Insulin attenuates LPS‐induced cognitive impairment and ferroptosis through regulation of glucose metabolism in hippocampus

**DOI:** 10.1111/cns.14887

**Published:** 2024-07-28

**Authors:** Miao Sun, Min Liu, Qingxiao Li, Siyuan Liu, Huikai Yang, Yuxiang Song, Mengyao Qu, Xiaoying Zhang, Yulong Ma, Weidong Mi

**Affiliations:** ^1^ Department of Anesthesiology The First Medical Center of Chinese PLA General Hospital Beijing China; ^2^ Department of Anesthesiology, Beijing Tongren Hospital Capital Medical University Beijing China; ^3^ Department of Nuclear Medicine The First Medical Center of Chinese PLA General Hospital Beijing China

**Keywords:** cognitive impairment, ferroptosis, insulin resistance

## Abstract

**Aims:**

Neuroinflammation is a recognized contributor to cognitive disorders like Alzheimer's disease, with ferroptosis emerging as a novel mechanism underlying cognitive dysfunction associated with neuroinflammation. Insulin, pivotal in the central nervous system, holds promise for cognitive function enhancement. This study aimed to establish a cognitive impairment model through intracerebroventricular injection of lipopolysaccharide (LPS) and explore the impact of intracerebroventricular insulin injection on cognitive function in mice.

**Methods:**

We employed diverse experimental techniques, including animal behavior testing, molecular assays, targeted metabolomics, nuclear medicine, and electron microscopy, to assess neurodegenerative changes, brain insulin resistance (IR), glucose uptake and metabolism, and ferroptosis. The model of cognitive impairment was induced via intracerebroventricular injection of LPS, followed by intracerebroventricular administration of insulin to evaluate its effects.

**Results:**

Insulin treatment effectively mitigated LPS‐induced cognitive decline and safeguarded against neuronal degeneration. Furthermore, insulin alleviated LPS‐induced insulin resistance, enhanced glucose uptake in the hippocampus, and promoted the Pentose Phosphate Pathway (PPP) and nicotinamide adenine dinucleotide phosphate (NADPH) production. Additionally, insulin activated the glutathione (GSH)‐glutathione peroxidase 4 (GPX4) pathway, reducing lipid peroxidation, and mitochondrial damage characteristic of LPS‐induced ferroptosis in the hippocampus.

**Conclusion:**

Our findings underscore the therapeutic potential of insulin in alleviating LPS‐induced cognitive impairment and ferroptosis by modulating glucose metabolism. This study offers a promising avenue for future interventions targeting cognitive decline.

## Graphical abstract

Insulin improves LPS‐induced cognitive impairment and alleviates LPS induced ferroptosis by promoting pentose phosphate pathway of glucose metabolism.

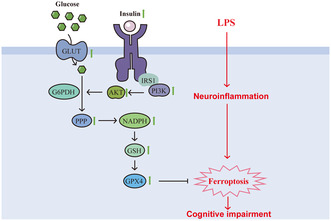

## 1 INTRODUCTION

Neuroinflammation, characterized by the activation of glial cells and the release of pro‐inflammatory factors in the central nervous system (CNS), has emerged as a significant contributor to the pathogenesis and progression of cognitive disorders such as aging, Alzheimer's disease (AD), and perioperative cognitive disorders (PND).^1^ The potential mechanisms through which neuroinflammation induces cognitive dysfunction encompass neurotoxicity, synaptic dysfunction, disruption of neurotransmitter systems, oxidative stress, and mitochondrial dysfunction.^2^ However, the pathological mechanisms underlying cognitive impairments caused by neuroinflammation remain incompletely understood.

Insulin serves as a critical signaling molecule in the brain, influencing synaptic plasticity, neuronal growth, survival, and regulating energy metabolism.^3^ Brain insulin resistance (IR), characterized by reduced responsiveness to insulin effects, results in a relative insufficiency of brain insulin levels. Brain IR has emerged as a significant factor contributing to cognitive impairment in conditions, such as AD and type 2 diabetes mellitus (T2DM).^3^ Furthermore, mounting evidence suggests that neuroinflammation can contribute to the development of brain IR.^4^ Inflammatory molecules, including cytokines and chemokines, disrupt insulin‐signaling pathways in the brain, impairing insulin's ability to regulate glucose uptake, metabolism, and synaptic plasticity.^4^ Therefore, brain IR represents a critical pathological factor through which neuroinflammation mediates cognitive impairment, although the underlying mechanism remains largely unknown.

Ferroptosis, a novel form of regulated cell death, is characterized by excessive lipid oxidation stress mediated by free iron, resulting in structural disruption of cell and mitochondrial membranes.^5^ Research has demonstrated the involvement of iron accumulation and ferroptosis injuries in brain tissues during various neurodegenerative processes.^5^ Our previous research has also revealed disrupted cerebral iron metabolism and ferroptosis‐associated damage in an animal model of cognitive dysfunction induced by LPS.^6^, ^7^ Administration of an iron chelator and ferroptosis inhibitor resulted in significant improvements in cognitive function and attenuation of cerebral ferroptosis.^6^ Therefore, ferroptosis emerges as a crucial pathological mechanism underlying neuroinflammation‐mediated cognitive impairment. However, limited research exists on the impact of brain insulin resistance on iron‐mediated cell death and whether insulin therapy can ameliorate ferroptosis during neuroinflammation‐induced cognitive dysfunction.

In this research, we induced an animal model of cognitive dysfunction through a single intracerebroventricular injection of LPS.^6^, ^7^ Employing a combination of experimental techniques, including behavioral tests, molecular assays, targeted metabolomics, nuclear medicine techniques, and electron microscopy, we assessed the effects of LPS on cognitive function, neurodegenerative changes, brain insulin resistance, glucose uptake and metabolism, ferroptosis‐related damage, as well as the therapeutic effects of insulin treatment in mice. This study aims to explore the neuroprotective effects of central insulin and provide novel insights and evidence for the prevention and treatment of clinical cognitive impairments.

## 2 MATERIALS AND METHODS

### 2.1 Animal treatments and experimental design

Approval for all experimental procedures was obtained from the Ethics Committee for Animal Experimentation at the Chinese PLA General Hospital. Male C57BL/6 mice, aged 8–10 weeks (18–20 g), were procured from the Chinese PLA General Hospital Laboratory Animal Center. They were housed in groups of 4–5 mice per cage, under standard conditions of constant temperature (22 ± 3°C) and humidity (50%–70%) with a 12‐h light/dark cycle. Mice were acclimatized for 1 week with ad libitum access to food and water before experimental interventions.

The cognitive impairment model was induced through intracerebroventricular injection of LPS, following established protocols.^7^ LPS (Sigma, USA) was dissolved in artificial cerebrospinal fluid (BIOFOUNT, China) to a 1 mg/mL LPS solution. Insulin (HumulinR, USA) was added to create the LPS + Insulin solution with an insulin concentration of 1 IU/mL.

A total of 99 mice (*n* = 33 per group) were categorized into three groups: Group S (3 μL artificial cerebrospinal fluid), Group L (3 μL LPS solution), and Group L + I (3 μL LPS + Insulin solution).

### 2.2 Morris water maze (MWM)

The MWM test was conducted as previously described.^7^ Each mouse underwent four trials per day over a 5‐day acquisition training period. On the 6th day, intracerebroventricular injection surgery was performed, and a probe test occurred 24 h post‐surgery. During the probe test, each mouse had 60 s to locate the removed platform. The average latency (s) in training trials, time percentage (%) in the target quadrant, target entries, and mean swimming speed (cm/s) during the probe test were recorded. All data were collected and analyzed by blinded researchers.

### 2.3 Y maze

The Y maze, assessing hippocampal‐dependent spontaneous spatial recognition, consisted of three arms (45 cm each) with a 120° intersection angle and visual cues at arm ends. The protocol, as reported^8^ with modifications, involved an acquisition phase with one arm closed 24 h post‐surgery. After a 2‐h interval, all arms were opened, and mice allowed to explore for 5 min. The time percentage (%) spent in the novel arm and the entries into the novel arm (defined as all limbs entering) were recorded. Start and closed/novel arms were randomly assigned using Excel, and data analysis was performed by blinded researchers.

### 2.4 Immunofluorescence staining

Immunofluorescence staining procedures were conducted following established protocols.^7^ Brains were harvested 24 h post‐surgery, subjected to perfusion with 0.9% pre‐cooled saline, and fixed with 4% paraformaldehyde. Coronal brain sections (10 μm) encompassing the entire hippocampus (*n* = 3) were meticulously prepared using a Leica CM1900 frozen slicer. Primary antibodies targeting GLUT3 (1:50, Santa Cruz Biotechnology, USA) and secondary antibodies (1:300, donkey anti‐mouse), labeled with Alexa‐594 (red, Invitrogen), were utilized. Samples were imaged with a fluorescence microscope (BX51; Olympus, Tokyo, Japan), and analysis performed by an observer blinded to the experimental groups.

### 2.5 TUNEL and FJB staining

TdT‐mediated dUTP nick end labeling (TUNEL) and Fluoro‐Jade B (FJB) staining of the hippocampus were performed 24 h post‐surgery according to the manufacturer's guidelines for the in situ Cell Death Detection Kit (Roche Applied Science, Germany) and FJB Powder for identifying degenerating neurons (Merck‐Millipore, USA), respectively, as previously described.^9^ Frozen brain sections (*n* = 3) were prepared as described earlier. Staining images were acquired using a fluorescence microscope, and the numbers of TUNEL‐positive and FJB‐positive cells in the hippocampal region were recorded and analyzed by an observer blinded to the experimental groups.

### 2.6 Western blot analysis

Mice were humanly euthanized by cervical decapitation under general anesthesia at the corresponding time point, followed by transcardial perfusion with pre‐cooled phosphate‐buffered saline (PBS). Brains were removed, washed in saline on ice, and hippocampi were carefully dissected and frozen in liquid nitrogen. Bilateral hippocampi were lysed together and subjected to western blot analysis as previously described.^7^ Antibodies utilized included anti‐P‐PI3K (1:1000, Abcam, USA), anti‐PI3K (1:1000, Abcam, USA), anti‐P‐AKT (1:1000, Abcam, USA), anti‐AKT (1:1000, Abcam, USA), anti‐P‐IRS1‐Ser612 (1:1000, CST, USA), anti‐IRS1 (1:1000, CST, USA), anti‐GLUT1 (1:1000, Abcam, USA), anti‐GLUT3 (1:1000, Abcam, USA), anti‐GLUT4 (1:1000, Abcam, USA), anti‐GAPDH (1:1000, Abcam, USA), and anti‐Caspase 3 (1:1000, Abcam, USA). Imaging was performed using the LI‐COR Odyssey System (LI‐COR Biotechnology, USA), and data were quantified with NIH Image J software. Band intensity analysis was conducted by experimenters blinded to the experimental groups.

### 2.7 Determination of GSH, NADPH, LPO, and MDA levels

To assess differences in ferroptosis‐related oxidative stress 24 h post‐surgery among the three groups, levels of GSH, NADPH, LPO, and MDA were measured using respective commercial assay kits (Nanjing Jiancheng, China) following the manufacturer's instructions. Results were normalized to total protein concentrations.

### 2.8 Positron emission tomography (PET) and biodistribution

PET was employed to assess glucose absorption and distribution in the brains of mice across different groups. The synthesis of ^18^F‐FDG, conducted by the Department of Nuclear Medicine at the PLA General Hospital, achieved a radiochemical purity (RCP) exceeding 99%. Mice (*n* = 3) received a 0.1 mL injection (200 μCi) of ^18^F‐FDG via tail veins 24 h post‐surgery. PET scans were conducted after 30 min using the SIGNA PET/MR scanner (Antpedia, China), with 1.5% isoflurane (RWD Life Science Co, USA) maintaining general anesthesia during scanning. A 10‐min PET scan and a 5‐min CT scan were performed for each mouse. Fusion images of PET and CT of the mouse brains were reconstructed on the Recon/Avator‐s‐10 workstation. The maximum standardized uptake value (SUVmax) of the region of interest (ROI), defined as the medial temporal lobe area, was recorded. Scanning and analysis procedures were executed by a radiologist blinded to the groups.

Biodistribution studies involved measuring the radioactive value of ^18^F‐FDG in the hippocampi 24 h post‐surgery (*n* = 5), with data adjusted with a time attenuation correction (%ID/g). Thirty minutes after injecting 0.1 mL (20 μCi) of ^18^F‐FDG through tail veins, mice were anesthetized, euthanized by cervical decapitation, and hippocampal tissues were collected, weighed, and assayed with a γ‐counter (Hidex, German). Results were calculated by a radiologist blinded to the experimental groups.

### 2.9 Targeted metabolomics analysis

Metabolomic analysis targeting glucose metabolism was conducted by LC‐Bio Technology Co., Ltd. (Hangzhou, China). Hippocampi (*n* = 3) were collected 24 h post‐surgery, and metabolomic extracts from 50 mg samples were analyzed using LC‐MS.^10^ Data analysis involved quality control, statistical analysis, selection of differential metabolites, and pathway enrichment analysis.

### 2.10 Transmission electron microscopy (TEM)

Mice (*n* = 3) were anesthetized and tissues fixed through intracardial perfusion with cold PBS containing 2.5% glutaraldehyde 24 h post‐surgery. Hippocampi were collected, and 1 × 1 × 1 mm tissue samples were prepared for TEM examination as described previously.^11^ Mitochondrial structures were assessed by an observer blinded to the groups, calculating the percentage of mitochondria exhibiting ferroptosis features.

### 2.11 Measurement of mitochondrial membrane potential (MMP)

Mitochondrial membrane potential (MMP), reflecting mitochondrial function, was measured using 5,5′,6,6′‐Tetrachloro‐1,1′,3,3′‐tetraethyl‐imidacarbocyanine iodide (JC‐1) staining (*n* = 3). Fresh hippocampal tissue samples were isolated rapidly after general anesthesia. Mitochondria were collected using a Tissue Mitochondria Isolation Kit (Beyotime, China). MMP levels were determined by the JC‐1 Mitochondrial Membrane Potential Assay Kit (Beyotime, China) and investigated using a fluorescence microplate reader (Thermofisher Scientific, USA). The ratio of aggregate/monomer fluorescent intensity was calculated to determine the level of mitochondrial membrane potential.^12^

### 2.12 Statistical analysis

All data were presented as mean ± standard error of the mean (SEM). MWM data, including escape latency, swimming speed, and swimming distance, underwent a two‐way repeated‐measures analysis of variance (ANOVA). Other data were analyzed using a one‐way ANOVA. Subsequent multiple comparisons were performed using either the Tukey test (assuming equal variances). The Shapiro–Wilk normality test was conducted to assess data distribution with SPSS 26.0 and the data which do not exhibit a normal distribution were analyzed via Kruskal–Wallis *H* and Dunn's multiple comparisons test. The statistical significance was set at *p <* 0.05. Analysis was performed with GraphPad 8.0 software.

## 3 RESULTS

### 3.1 Insulin treatment markedly improved LPS‐induced cognitive impairment

**FIGURE 1:**
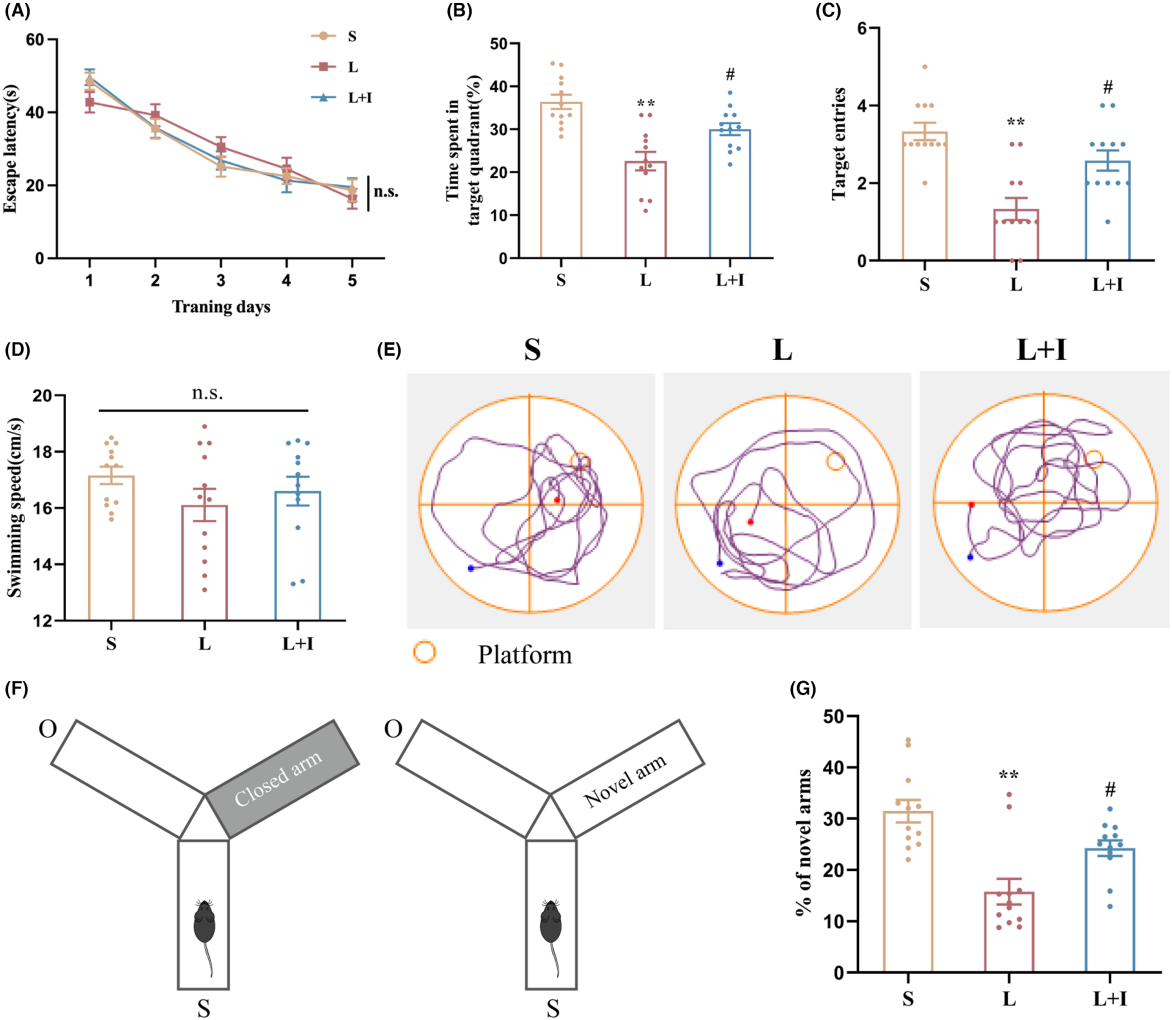
Insulin treatment alleviates LPS‐induced cognitive impairment. Results from Morris water maze (MWM) test (*n* = 12): (A) Average escape latency (s) during the 5‐day training period. (B) Percentages of time (s) spent by mice in the target quadrant during the probe test. (C) Number of times mice crossed the target locations in the probe. (D) Average swimming speed (cm/s) of mice in the probe test. (E) Representative track images of swimming pathways for the three groups in the probe test. Results from Y maze (*n* = 12): (F) Schematic diagram of the Y maze process. (G) Percentages of time (%) spent in novel arms. Data are presented as means±SEM. **p < 0.01 vs. Group S, and ^#^ *p* < 0.05 vs. Group L. MWM, Morris water maze. n.s., not significant. S, Group S; L, Group L; L + I, Group L + I.

The MWM and Y maze assessments were used to evaluate the protective efficacy of insulin treatment against hippocampus‐dependent cognitive impairment induced by LPS. During the training period, mice across experimental groups exhibited comparable escape latency times (Figure 1A). After LPS administration, the probe test revealed significant cognitive impairment in LPS‐treated animals (Group L) compared to the controls treat with artificial cerebrospinal fluid (Group S), evidenced by reduced platform crossings (1.33 ± 0.28, **p < 0.01 vs. Group S) and diminished time spent in the target quadrant (22.58 ± 2.15%, **p < 0.01 vs. Group S) (Figure 1B,C). Notably, insulin treatment (Group L + I) significantly ameliorated cognitive deficits, increasing time spent in the target quadrant (30.03 ± 1.39%, ^#^ *p* < 0.05 vs. Group L) and the frequency of entries into the target location (2.58 ± 0.26, ^#^ *p* < 0.05 vs. Group L) during probe trials. These results indicate that LPS‐induced cognitive decline was effectively reversed by insulin treatment. Representative swimming pathways in Figure 1E illustrate the distinct behavioral patterns of the three groups, further confirming the beneficial effects of insulin. Average swimming speeds among groups (Figure 1D) remained comparable (17.16 ± 0.31, 16.11 ± 0.57, 16.60 ± 0.51 cm/s in Group S, L, and L + I, respectively), indicating consistent motor functions post‐surgery.

In the Y maze assessment (Figure 1F,G), conducted 24 h after surgery, mice in Group L demonstrated significantly less time exploring novel arms compared to Group S (15.76 ± 2.51% vs. 31.47 ± 2.20%, *p* < 0.01). Insulin treatment in Group L + I notably increased the time spent in novel arms (24.26 ± 1.53%, ^#^ *p <* 0.05 vs. Group L), suggesting a robust improvement in spatial memory. Collectively, these results underscore the profound cognitive protective effects of insulin against LPS‐induced impairment.

### 3.2 Insulin treatment significantly decreased LPS‐induced neurotoxicity in the hippocampus

**FIGURE 2:**
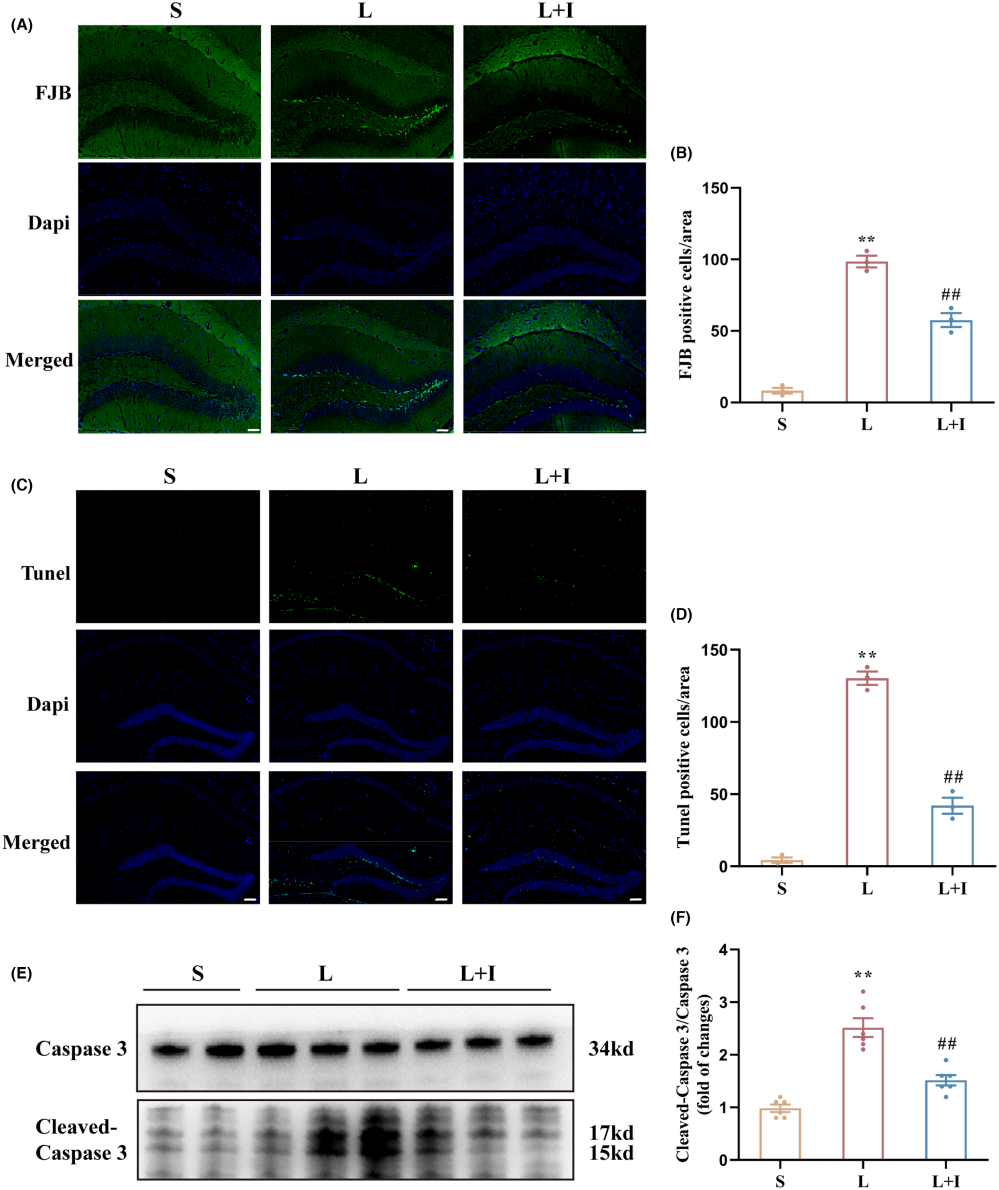
Insulin treatment improves LPS‐induced neural damage. (A) Representative images of Fluoro‐Jade B (FJB) staining in the hippocampus 24 h after surgery (*n* = 3). (B) Statistical analysis of FJB‐positive cells in the three groups. (C) TdT‐mediated dUTP nick end labeling (TUNEL) staining of the hippocampus to visualize neural apoptosis in the different groups (*n* = 3). (D) Statistical analysis of TUNEL‐positive cells in the three groups. (E) Representative images showing the expression of Iba1 and GFAP proteins in the hippocampus 24 h after surgery (*n* = 6). (F) Graph depicting relative expression levels of Caspase 3 and Cleaved‐Caspase 3 in the hippocampus 24 h after surgery. Scale bars = 100 μm. Data are presented as means±SEM. **p < 0.01 vs. Group S, and ^##^ *p* < 0.01 vs. Group L. S, Group S; L, Group L; L + I, Group L + I. FJB, Fluoro‐Jade B; TUNEL, TdT‐mediated dUTP nick end labeling.

To assess the levels of LPS‐induced neuronal degeneration in the hippocampus, we next performed Fluoro‐Jade B (FJB) staining in fixed tissue samples (Figure 2A). As depicted in Figure 2B, the presence of FJB‐positive cells significantly increased in Group L compared to Group S (98.67 ± 4.06 vs. 8.33 ± 2.03, *p* < 0.01). Notably, insulin treatment effectively reduced the number of FJB‐positive cells in the hippocampal regions (57.67 ± 4.91, *p* < 0.01 vs. Group L), indicating a significant mitigation of neuronal degeneration.

TUNEL staining was then utilized to assess neuronal apoptosis within the hippocampus (Figure 2C). The quantification in Figure 2D revealed a total of 4.33 ± 1.86, 130.30 ± 4.63, and 42.00 ± 5.51 TUNEL‐positive cells in Group S, L, and L + I, respectively. LPS administration significantly increased the number of TUNEL‐positive cells in Group L compared to Group S (*p <* 0.01). Conversely, insulin treatment in Group L + I substantially decreased the number of TUNEL‐positive cells (^##^ *p* < 0.01 vs. Group L), indicating a significant attenuation of neuronal apoptosis.

To further delineate the extent of neuronal apoptosis in the hippocampus, western blot analysis was conducted to assess the relative expression levels of cleaved‐caspase 3 (Figure 2E). The expression levels of cleaved‐caspase 3/caspase 3 in Group L were significantly higher than in Group S (*p* < 0.01). Notably, insulin treatment in Group L + I markedly decreased the expression levels of cleaved‐caspase 3/caspase 3 (^#^ *p* < 0.05 vs. Group L) 24 h after surgery (Figure 2F), reinforcing the neuroprotective role of insulin against LPS‐induced apoptosis in the hippocampus.

### 3.3 Insulin treatment dramatically promoted glucose uptake by alleviating insulin resistance in hippocampus

**FIGURE 3:**
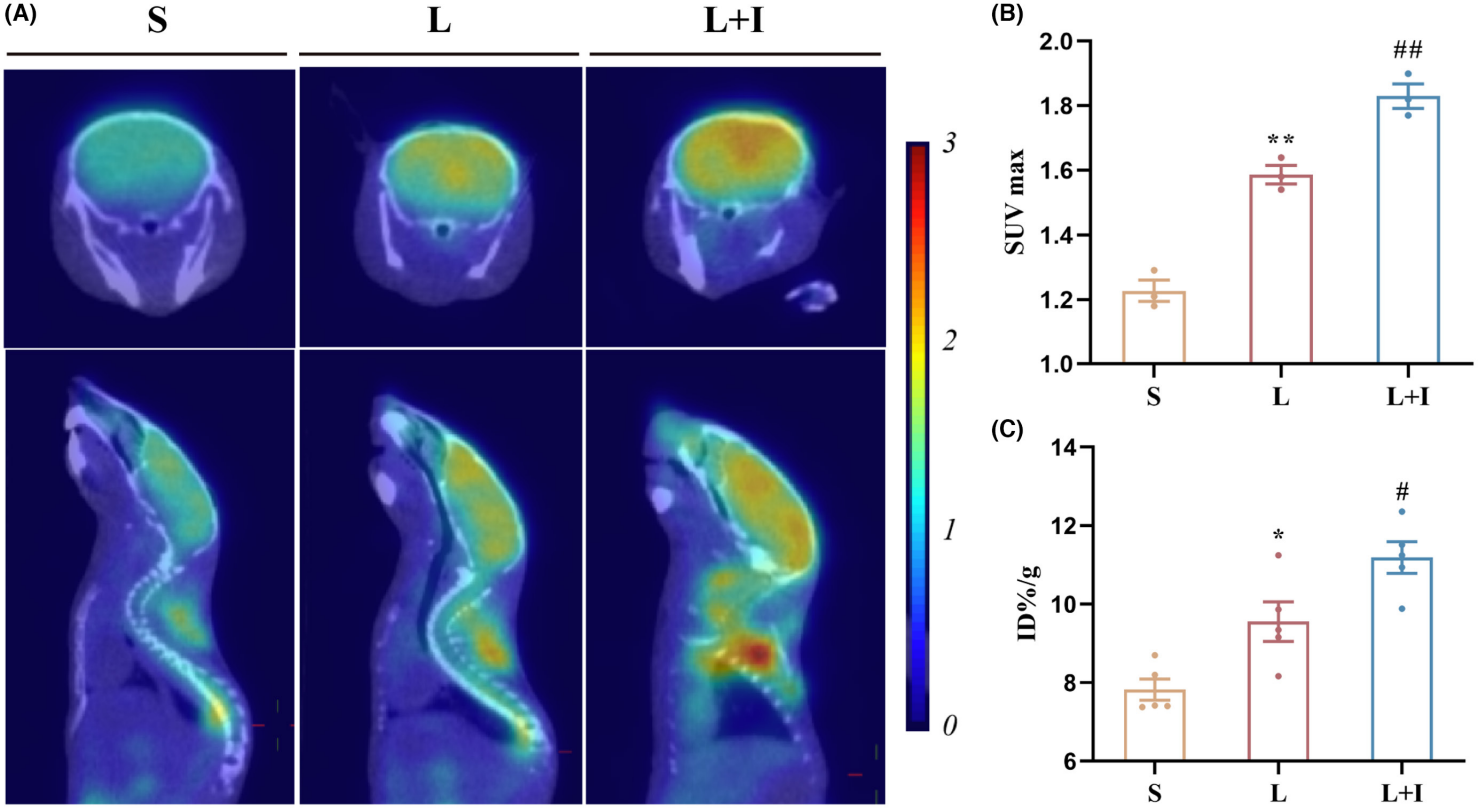
Insulin treatment promoted glucose uptake in hippocampus. (A) Representative PET images of ^18^F‐FDG in different sections (*n* = 3). (B) Maximum standardized uptake value (SUVmax) of ^18^F‐FDG in brains from different groups. (C) Quantification of ^18^F‐FDG biodistribution in the hippocampus of different groups 24 h after surgery (*n* = 5). Data are presented as means ± SEM. *p < 0.05, **p < 0.01, vs. Group S, and ^##^ *p* < 0.01, ^#^ *p* < 0.05 vs. Group L. S, Group S; L, Group L; L + I, Group L + I. ID%/g, percentage injected dose per gram; SUVmax, maximum standardized uptake value.

To evaluate the impact of LPS on hippocampal glucose uptake, we conducted ^18^F‐FDG‐PET and biodistribution studies. Figure 3A illustrates the use of ^18^F‐FDG as a probe for PET scanning to reflect glucose uptake in the brains. The standardized uptake values (SUVmax) were 1.23 ± 0.03, 1.59 ± 0.03, and 1.83 ± 0.04 in Groups S, L, and L + I, respectively (Figure 3B). LPS significantly increased the uptake of ^18^F‐FDG (**p < 0.01, vs. Group S). Importantly, insulin treatment further elevated ^18^F‐FDG uptake in hippocampal regions (^#^ *p* < 0.05, vs. Group L), demonstrating the potent ability of insulin to promote glucose uptake.

To quantify ^18^F‐FDG content in the hippocampus, biodistribution analysis was performed (Figure 3C). The ^18^F‐FDG levels were 7.82 ± 0.27 ID%/g, 9.55 ± 0.51 ID%/g, and 11.19 ± 0.40 ID%/g in Groups S, L, and L + I, respectively. LPS administration significantly increased the uptake of ^18^F‐FDG (*p* < 0.01), while insulin treatment further enhanced ^18^F‐FDG uptake in the hippocampus (^#^ *p* < 0.05, vs. Group L).

**FIGURE 4:**
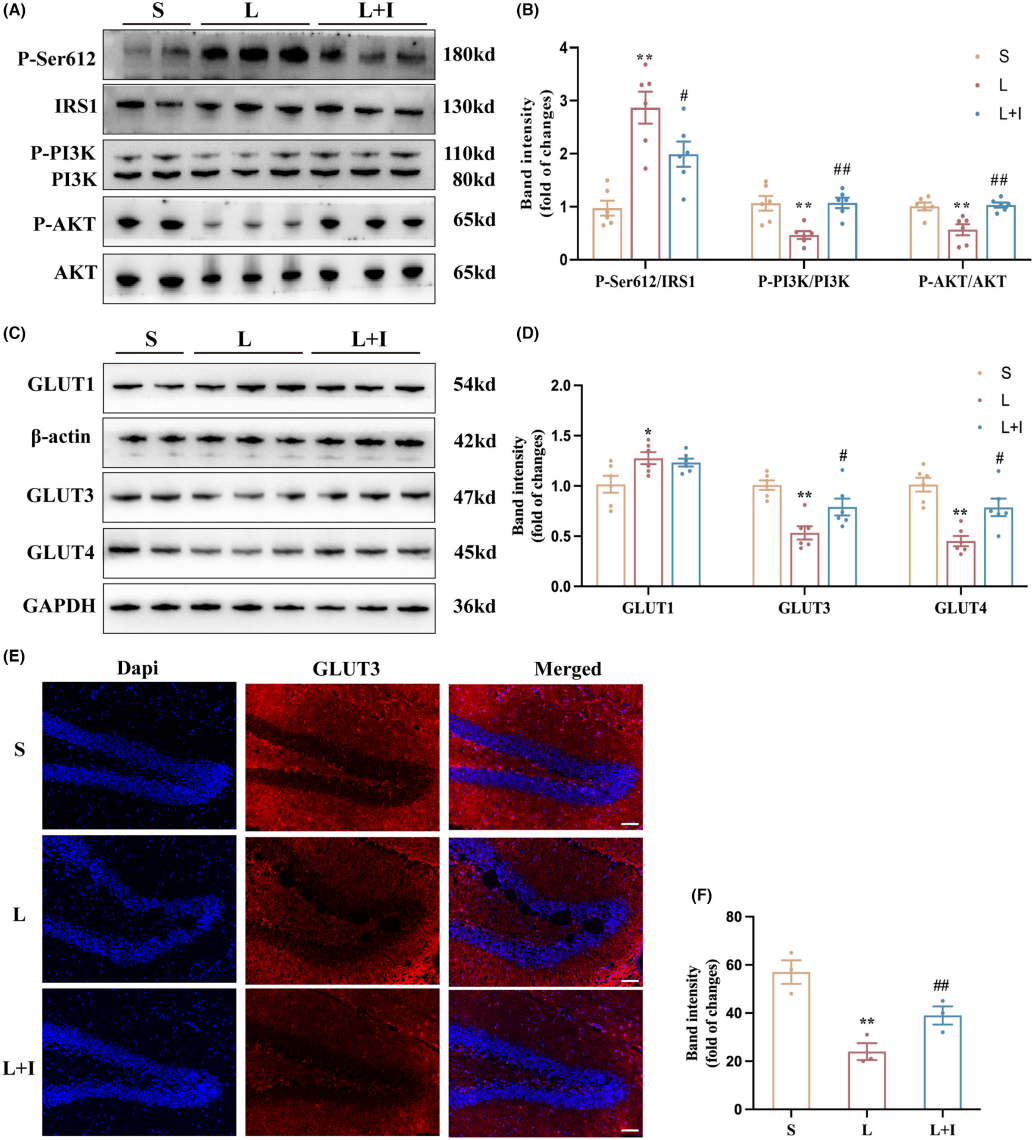
Insulin treatment modulates the expression of IR‐related proteins and GLUTs. (A) Western blot analysis of insulin signaling pathway components (P‐Ser612/IRS1, PI3K, P‐PI3K, AKT, P‐AKT) and glucose transporters (GLUT1, GLUT3, GLUT4) in the hippocampus 24 h after surgery. (B) Quantitative analysis of the expression levels of P‐Ser612/IRS1, PI3K, P‐PI3K, AKT, P‐AKT in different groups. (C) Quantification of GLUT1, GLUT3, and GLUT4 expression levels in different groups. (D) Representative images of western blots. Data are means ± SEM. (E) Representative images of immunofluorescence staining of GLUT3 in the hippocampus 24 h after surgery (*n* = 3). (F) Statistical analysis of GLUT3‐positive neurons in the three groups (*n* = 3). *p < 0.05, **p < 0.01 vs. Group S, and ^##^ *p <* 0.01, ^#^ *p <* 0.05 vs. Group L. S, Group S; L, Group L; L + I, Group L + I. AKT, protein kinase B; GLUT, glucose transporter; P‐AKT, phosphorylated AKT; PI3K, phosphoinositide 3‐kinase; P‐PI3K, phosphorylated PI3K; P‐Ser612/IRS1, phosphorylated Ser612 on IRS1.

Western blot analysis was employed to investigate the effects of LPS administration and insulin treatment on insulin resistance (IR) in the hippocampus (Figure 4A). As depicted in Figure 4B, the expression levels of the IR biomarker P‐Ser612/IRS1 were significantly increased in Group L (**p < 0.01, vs. Group S), and insulin treatment markedly inhibited this trend induced by LPS (^#^ *p* < 0.05, vs. Group L).

Furthermore, expression levels of P‐PI3K/PI3K in Group L were significantly decreased (**p < 0.01, vs. Group S), while insulin treatment markedly increased the activation of PI3K in Group L + I (^##^ *p* < 0.01, vs. Group L). A similar trend was observed in the expression levels of P‐AKT/AKT, where LPS administration significantly inhibited the activation of AKT in Group L (**p < 0.01, vs. Group S). However, insulin treatment restored the expression levels of P‐AKT/AKT in Group L + I (^##^ *p* < 0.01, vs. Group L).

To provide insights into the mechanism of hippocampal glucose uptake influenced by LPS administration and insulin treatment, the expression levels of GLUT1, GLUT3, and GLUT4 were assessed by western blot (Figure 4C). As shown in Figure 4D, LPS administration significantly increased GLUT1 expression in Group L and Group L + I (*p < 0.05, vs. Group S), with no statistical difference between Group L and Group L + I. However, both the expression levels of GLUT3 and GLUT4 were inhibited in Group L (**p < 0.01, vs. Group S), and insulin treatment improved the expression levels of GLUT3 and GLUT4 in Group L + I (^#^ *p <* 0.05, vs. Group L), highlighting the role of insulin in modulating glucose transporters and mitigating insulin resistance‐induced impairment in hippocampal glucose uptake. Figure 4D,E presented the GLUT3 expression in hippocampus and the trends were consist with the results of western blot. LPS administration significantly decreased the expression levels of GLUT3 in Group L (**p < 0.01, vs. Group S). However, insulin treatment restored the expression levels of GLUT3 in Group L + I (^#^ *p* < 0.05, vs. Group L).

### 3.4 Insulin treatment promoted PPP in hippocampal glucose metabolism after LPS administration

**FIGURE 5:**
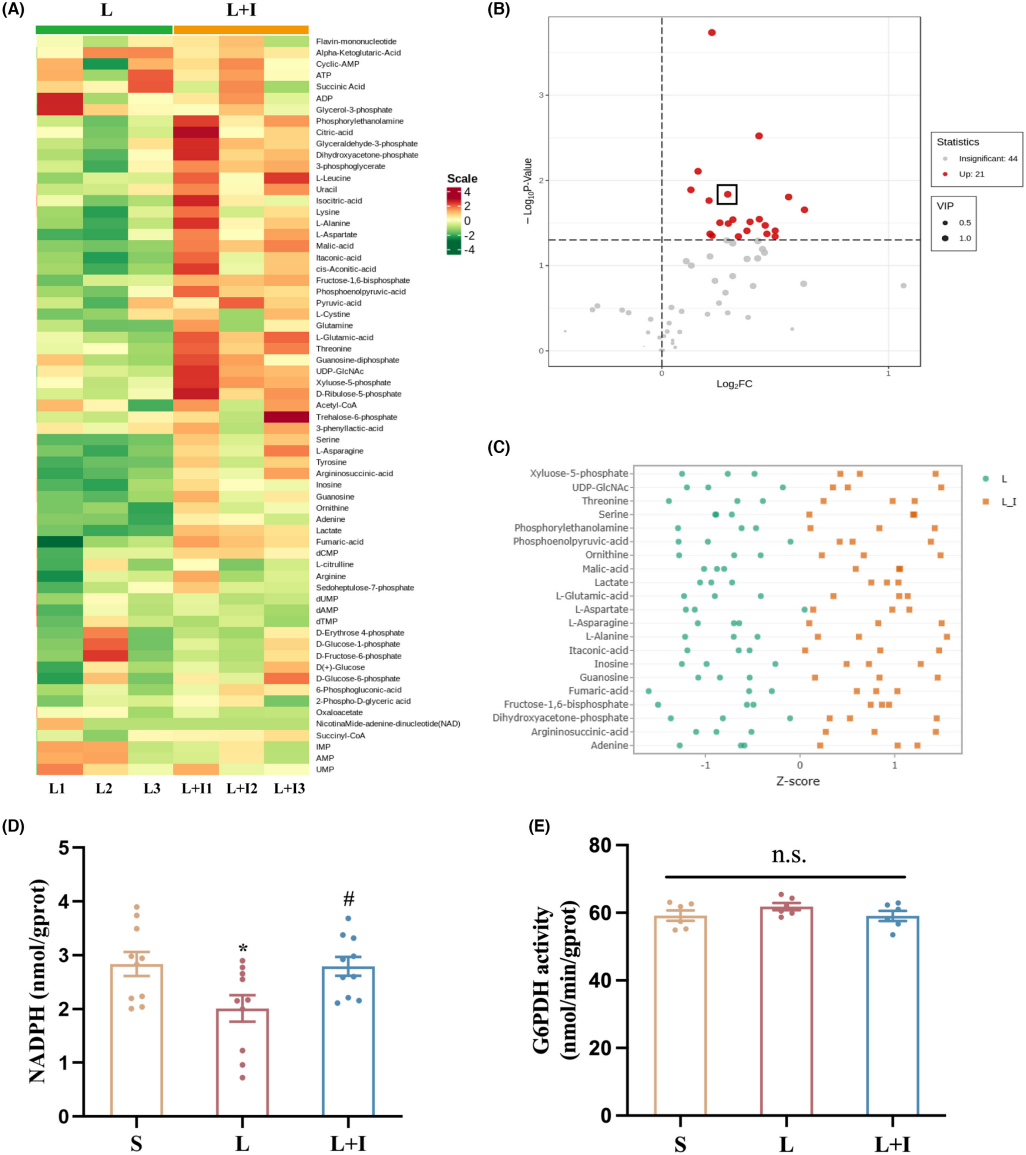
Insulin treatment promoted PPP and NADPH production in hippocampus. Results of targeted metabolomics analysis (*n* = 3): (A) Heat map depicting the metabolite profile in Group L and Group L + I. (B) Volcano plots illustrating differential metabolites, with red points representing significantly upregulated metabolites. The black box highlights the metabolite “ribose‐5‐phosphate.” The *x*‐axis indicates the fold change in metabolite abundance between groups, while the *y*‐axis indicates the significance level. (C) Normalized values of differential metabolites, emphasizing the elevation of ribose‐5‐phosphate in Group L + I. (D) Levels of nicotinamide adenine dinucleotide phosphate (NADPH) in the hippocampus 24 h after surgery (*n* = 6). (E) Activity of glucose‐6‐phosphate dehydrogenase (G6PDH) in the hippocampus of different groups (*n* = 6). Data are means ± SEM. *p < 0.05 vs. Group S, and ^#^ *p* < 0.05 vs. Group L. S, Group S; L, Group L; L + I, Group L + I. G6PDH, glucose‐6‐phosphate dehydrogenase; NADPH, nicotinamide adenine dinucleotide phosphate.

To unravel the effects of LPS administration and insulin treatment on hippocampal glucose metabolism, we next used targeted metabolomics analysis of glucose metabolism products on hippocampal tissue samples from Groups L and L + I. Figure 5A illustrates the detection of 65 metabolites showing noticeable differences in principal component analysis results between the two groups (Figure 5A). Following differential metabolite screening criteria (Figure 5B), 21 metabolites exhibited significant differences. Ribose‐5‐phosphate, a metabolite of the pentose phosphate pathway (PPP), was notably elevated in the L + I group (Figure 5C).

The study then assessed NADPH levels in the hippocampus (Figure 5D). The levels were 2.84 ± 0.22, 2.01 ± 0.25, and 2.80 ± 0.18 nmol/g.prot in Groups S, L, and L + I, respectively. LPS administration significantly decreased NADPH production in the hippocampus while insulin treatment reversed this trend.

Furthermore, we next measured the activity of glucose‐6‐phosphate dehydrogenase (G6PDH), a key enzyme in the PPP, by ELISA. However, as depicted in Figure 5E, there were no statistically significant differences in G6PDH activity among the three groups of hippocampal tissue, suggesting that central insulin does not promote hippocampal PPP metabolism by increasing G6PDH activity.

### 3.5 Insulin treatment inhibits LPS‐induced ferroptosis in the hippocampus

**FIGURE 6:**
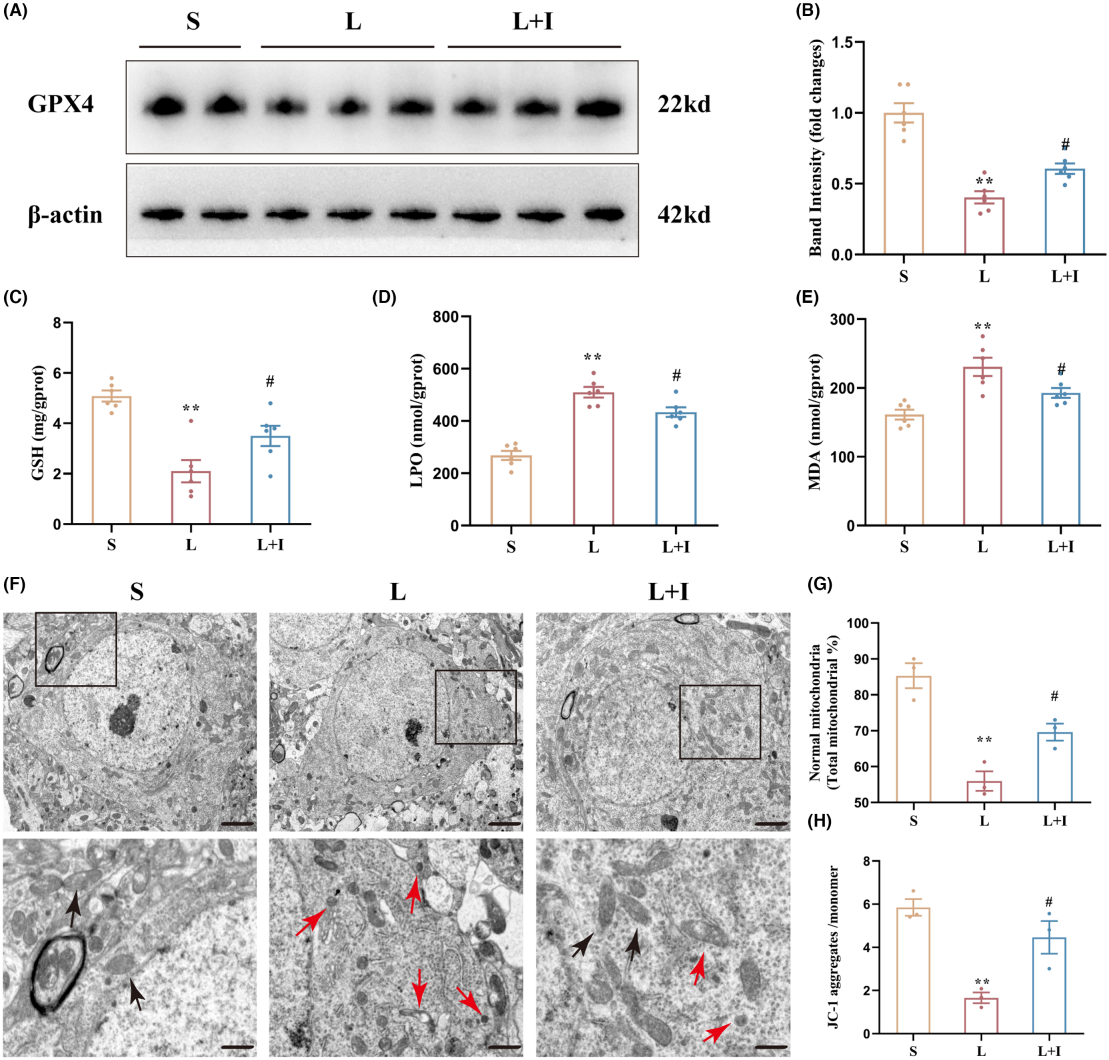
Insulin treatment alleviates LPS‐induced ferroptosis in hippocampus. (A) Representative images depicting the expression of GPX4 in the hippocampus 24 h after surgery (*n* = 6). (B) Graph illustrating the relative expression levels of GPX4 in the hippocampus 24 h after surgery. (C) Levels of glutathione (GSH) in the hippocampus 24 h after surgery (*n* = 6). (D) Levels of lipid peroxide (LPO) in the hippocampus 24 h after surgery (*n* = 6). (E) Levels of malondialdehyde (MDA) in the hippocampus 24 h after surgery (*n* = 6). (F) Representative electron microscopy images displaying mitochondrial morphology in the hippocampus 24 h after surgery (*n* = 3). Scale bars = 2 μm (top) and 100 nm (bottom). Black arrows indicate normal mitochondria with contact structure and clear inner ridges, while red arrows represent abnormal mitochondria with reduced volume and dense inner ridges. (G) Quantitative analysis of the percentage of normal mitochondria. (H) Quantitative analysis of JC‐1 aggregates/monomer in different groups (*n* = 3). Data are means ± SEM. **p < 0.01 vs. Group S and ^#^ *p* < 0.05 vs. Group L. S, Group S; L, Group L; L + I, Group L + I. GPX4, glutathione peroxidase 4; GSH, glutathione; JC‐1, 5,5′,6,6′‐Tetrachloro‐1,1′,3,3′‐tetraethyl‐imidacarbocyanine iodide; LPO, lipid peroxide; MDA, malondialdehyde.

The expression levels of the key ferroptosis‐related protein GPX4 were evaluated by western blot (Figure 6A). Quantitative analysis indicated that the expression levels of GPX4 in Group L were significantly decreased relative to Group S (*p* < 0.01), while insulin treatment markedly increased GPX4 in Group L + I compared with Group L (*p* < 0.05).

To investigate the protective effect of insulin treatment on hippocampal oxidative stress induced by LPS, GSH, LPO, and MDA levels were measure by ELISA. GSH levels were 5.08 ± 0.22, 2.10 ± 0.44, and 3.50 ± 0.40 mg/g.prot in Groups S, L, and L + I, respectively (Figure 6C). LPS administration significantly decreased GSH levels in the hippocampus (**p < 0.01, vs. Group S), and insulin treatment markedly alleviated the decrease of GSH levels induced by LPS (^#^ *p* < 0.05, vs. Group L).

As shown in Figure 6D, LPO levels were 268.30 ± 17.61, 509.80 ± 20.39, and 433.90 ± 18.51 nmol/g.prot in Groups S, L, and L + I, respectively. LPS administration promoted hippocampal LPO production (**p < 0.01, vs. Group S), and insulin treatment markedly decreased LPO production in Group L + I (^#^ *p* < 0.05, vs. Group L). Similar results were obtained for MDA levels (Figure 6E), where LPS administration significantly increased hippocampal MDA levels in Group L (230.70 ± 13.28 mmol/g.prot) relative to Group S (161.30 ± 7.11 mmol/g.prot, *p* < 0.01). Again, insulin treatment markedly decreased the MDA levels in Group L + I (192.80 ± 7.11 mmol/g.prot, ^#^ *p* < 0.05, vs. Group L).

Transmission electron microscopy was used to investigate mitochondrial structures in the three groups (Figure 6F). Mitochondria in Group S showed outer membranes of normal size and shape, while abnormal mitochondrial morphology, characteristic of ferroptosis, was observed in Groups L and L + I, including shrunken size with condensed mitochondrial membrane densities. Mitochondria crista was decreased or absent, and outer mitochondrial membranes were broken. However, cells in Group L + I had less abnormal mitochondria. The percentages of normal mitochondria were 85.33 ± 3.49%, 55.97 ± 2.72%, and 69.60 ± 2.39% in Groups S, L, and L + I, respectively (Figure 6G). The percentage of normal mitochondria in Group L was remarkably lower than in Group S (**p < 0.01), and Group L + I (^#^ *p* < 0.05) showed a significant improvement. The ratios of JC‐1 aggregates to JC‐1 monomers were 5.85 ± 0.39, 1.66 ± 0.25, and 4.46 ± 0.76 in Groups S, L, and L + I, respectively, as shown in Figure 6H. LPS administration significantly induced the decline of mitochondrial membrane potential (MMP) (**p < 0.01, vs. Group S), but insulin treatment markedly reversed this trend (^#^ *p* < 0.05, vs. Group L), indicating the protective effect of insulin against LPS‐induced ferroptosis in the hippocampus.

## 4 DISCUSSION

Neuroinflammation is an intricate process initiated by immune cell activation in the brain in response to injury, infection, or neurological stimuli that releases pro‐inflammatory molecules, such as cytokines and reactive oxygen species, contributing to neuronal death and cognitive decline.^1^, ^2^ Our study utilized LPS, a component of Gram‐negative bacteria outer membrane, to induce neuroinflammation, replicating a model of cognitive dysfunction.^6^, ^7^ This model successfully triggered cognitive impairment, neuronal degeneration, and apoptosis. Strikingly, insulin treatment demonstrated a significant improvement in cognitive function and an increase in the survival of neurons in the hippocampus. The neuroprotective mechanism of insulin in managing inflammatory disorders remains elusive.

Insulin exerts diverse functions in the brain, encompassing the regulation of glucose metabolism, promotion of neuronal survival, growth, and modulation of neurotransmitter levels.^3^ Its involvement in memory formation and learning underscores its pivotal role in brain function.^3^ Notably, brain insulin resistance (IR) emerges as a potential pathological mechanism mediated by neuroinflammation in cognitive impairment.^13^ Our study corroborates this by revealing LPS‐induced impairment of insulin signaling and increased IR biomarkers, subsequently ameliorated by insulin treatment. Impaired insulin signaling and reduced responsiveness are implicated in Alzheimer's disease (AD), mild cognitive impairment (MCI), and other neurodegenerative conditions.^3^, ^14^ The administration of intranasal insulin, as demonstrated in clinical studies, has proven effective in enhancing cognitive function across various cognitive disorders.^15^ Although our study opted for intracerebroventricular injection for insulin delivery, a method chosen for its stability and reliability, the specific mechanisms of insulin's neuroprotective effects in the context of neuroinflammation require further exploration.

Glucose, the brain's primary energy source, sustains essential processes, including neurotransmission and synaptic plasticity, crucial for cognitive functions.^16^ Dysregulation of glucose metabolism underlies brain IR in cognitive impairment.^17^ Our biodistribution study showed that insulin treatment significantly increased hippocampal glucose uptake, attributed to the activation of the PI3K‐AKT signaling pathway—a major insulin pathway for regulating glucose metabolism. Analysis using ^18^F‐FDG‐PET revealed a significant decrease in glucose uptake and metabolism rates within certain brain regions, including the hippocampus, frontal cortex, and parietal cortex, in individuals with mild cognitive impairment and AD. Additionally, decreased GLUT expression in the brain has been reported in AD patients.^18^ Consequently, maintaining glucose uptake and utilization in brain tissue is crucial for normal physiological function, and the neuroprotective effects of insulin may be linked to improved brain IR and increased brain glucose uptake.

Interestingly, LPS administration also increased glucose uptake, linked to upregulated GLUT1 expression associated with microglial activation,^19^ but downregulated GLUT3 and GLUT4 expression. Insulin treatment did not affect GLUT1 expression, but it significantly increased the expression levels of GLUT3 and GLUT4, which likely explain the strikingly neurological outcome. In the context of microglia activation, GLUT1 expression is upregulated, and increased expression and activity of GLUT1 facilitates the glucose uptake from the extracellular environment. This ensures adequate energy supply for the elevated metabolic demands of activated microglia.^19^ Our previous studies revealed over activation of microglia induced by LPS administration,^7^ therefore, up‐regulated expression of GLUT1 and increased glucose uptake in the present research was associated to microglia activation. The expression of GLUT3 is predominantly localized in neurons, particularly in regions of the brain that rely heavily on glucose metabolism, such as the cerebral cortex and hippocampus.^20^ This selective expression of GLUT3 allows neurons to maintain a constant supply of glucose, even under conditions of high energy demand.^20^ Expression of GLUT4 is regulated by the insulin signal pathway PI3K‐AKT and dysregulation of GLUT4 is reported to be involved in multiple CNS disorders like AD, Parkinson's disease and obesity‐related cognitive impairments.^18^ Together with our observations, these elucidate insulin's differential impact on GLUT expression in distinct neural cell types, and emphasize the need for future research to delve into insulin's cell‐specific effects in the CNS.

Targeted metabolomic analysis of glucose metabolites in the hippocampus revealed insulin's promotion of the pentose phosphate pathway (PPP) and NADPH production under neuroinflammatory conditions. Glucose metabolism in the brain primarily involves glycolysis, oxidative phosphorylation, the PPP, as well as glycogen, amino acid, and fatty acid synthesis.^21^ In neurons, the PPP contributes to NADPH generation for antioxidant defense,^22^ where it provides the reducing equivalents needed to keep GPX4 in its active reduced form, enabling neutralization of lipid peroxides and preventing cell death.^23^ Previous reports have connected G6PD activity to PPP metabolism and activator of PI3K signaling pathway.^24^ In our study, G6PDH activity remained unaffected, indicating insulin's influence on PPP via the substrate levels; further investigations are warranted to fully clarify this mechanism.

The PPP's role in antioxidant defense, nucleotide synthesis, and redox signaling underscores its significance in ferroptosis, a process linked to cognitive impairments.^22^ Ferroptosis, implicated in various cognitive impairments,^6^ has been effectively alleviated by central insulin treatment.^7^ Meanwhile, NADPH, a crucial determinant of redox homeostasis, plays a multifaceted role in ferroptosis by supporting antioxidant defenses and inhibiting lipid peroxidation.^23^ Additionally, low NADPH basal levels can serve as a biomarker of cell line sensitivity to ferroptosis.^25^ Our study demonstrated that insulin treatment preserved neuronal and mitochondrial function in the hippocampus against ferroptosis injuries. This indicates that insulin's inhibitory effect on LPS‐induced ferroptosis may be attributed to the maintenance of NADPH levels, representing a potential therapeutic avenue for cognitive dysfunction associated with neuroinflammation. Future research should delve into the specific mechanisms underlying insulin's modulation of ferroptosis in the CNS.

## 5 CONCLUSION

In conclusion, our study provides evidence that intracerebroventricular administration of insulin effectively attenuates LPS‐induced cognitive deficits, neuronal degeneration, and ferroptosis by enhancing glucose uptake and promoting the pentose phosphate pathway (PPP) in glucose metabolism. Our findings highlight insulin's multifaceted neuroprotective effects in the context of neuroinflammation‐induced cognitive impairment. The intricate interplay between insulin, glucose metabolism, and ferroptosis underscores the complexity of the mechanisms involved, but support the potential of central insulin as a therapeutic approach to preserve cognitive function in the context of neuroinflammation. As insulin emerges as a potential therapeutic target, future investigations should aim to unravel the detailed cellular and molecular pathways, paving the way for innovative treatments for neuroinflammation‐associated cognitive disorders.

## AUTHOR CONTRIBUTIONS

**Miao Sun:** Investigation; Methodology; Project administration; Writing original draft. **Min Liu:** Investigation; Methodology; Project administration. **Qingxiao Li:** Investigation; Methodology. **Siyuan Liu:** Project administration; Software. **Huikai Yang:** Data curation**. Yuxiang Song:** Project administration. **Mengyao Qu:** Data curation; Validation. **Xiaoying Zhang:** Data curation. **Yulong Ma:** Conceptualization; Funding acquisition. **Weidong Mi:** Conceptualization; Funding acquisition; Writing – review & editing.

## FUNDING INFORMATION

This work was financially supported by the National Natural Science Foundation of China (Nos. 82171180, 82371469, 82171464, 81901097).

## CONFLICT OF INTEREST STATEMENT

The authors have no conflict of interest to declare.

## Data Availability

The datasets generated during and/or analyzed during the current study are available from the corresponding author upon reasonable request.
